# Supplementary material for: Genetic influence is linked to cortical morphology in category-selective areas of visual cortex
Source: Nat Commun. 2020 Feb 5;11:709. doi: 10.1038/s41467-020-14610-8 (PMC7002610; doi:10.1038/s41467-020-14610-8)

Supplementary Information

Genetic influence is linked to cortical morphology in category-selective areas of visual cortex

Abbasi et al.

Supplementary Figure Captions:

Supplementary Figure 1: Spatial overlap between category-selective activations in MZ and DZ twins. In each subject, face-, body-, and place-related activation maps were thresholded at different z values (z threshold: 0 to 8 with steps of 0.5), and the resulting maps were binarized. For each twin pair, the spatial overlap between the binarized maps was calculated using Dice coefficient. The overlap coefficients were then averaged across all MZ and DZ twin pairs.

Supplementary Figure 2: Face-selective voxels in Amygdala. Left and right amygdala (blue patches) are shown on axial, coronal, and sagittal slices of an average ($n = 881$) anatomical T1 volume. Face-selective voxels (yellow patches) were concentrated in the centromedial subdivision (central and medial nuclei) of amygdala. Out of 36 face-selective voxels in left+right amygdala, 5 voxels showed a significant (uncorrected $p < 0.05$) genetic effect. After FDR correction, none of the voxels in amygdala showed a significant effect.

Supplementary Figure 3: Similarity of functional activation patterns in category-selective areas. Based on an analysis described in **Figure 1b**, the Pearson's r correlation was computed between the activation patterns in MZ twin pairs, DZ twin pairs, and unrelated pairs. Only face-, body-, and place-selective voxels (913 voxels for each category) were included in this analysis. Unrelated pairs were generated by shuffling subjects in the DZ group (similar results were obtained with shuffling subjects in the MZ group). In each category, the mean correlation varied significantly ($p < 0.0005$; one-way

ANOVA) across the three groups, and all pairwise comparisons were significant ($p < 0.05$; Tukey's HSD Post-Hoc test), except for the comparison of DZ twins versus unrelated pairs in the face category ($p > 0.05$).

Supplementary Figure 4: Path diagram of the genetic structural equation model. A, D, C, and E are latent factors. A represents additive genetic effects (the sum of the effects of individual alleles). D represents dominance genetic effects (interactions between alleles). C represents common environmental effects. E represents unique environmental effects and measurement error. Double arrows represent the correlations between twin pairs. Genetic effects are correlated 1 between MZ twins, as they share 100% of their genes. The correlations between DZ twins are 0.5 and 0.25 for A and D, respectively. Common environmental effects have correlation 1 for both types of twins, while unique environmental effects are uncorrelated (the correlation is expected to be 0). a, d, c, and e are regression path coefficients of the respective latent factors. Figure reproduced from **Tsagkrasoulis et al., 2017**.

Supplementary Figure 5: Histograms showing the distributions of h^2 and c^2 in face, body, and place areas.

Supplementary Figure 6: Cross-validation analysis confirming genetic influence maps in category-selective areas. For this analysis, data in MZ and DZ groups were randomly split into two independent halves. Genetic analysis was performed for both split-half samples (67 MZ twin pairs and 39 DZ twin pairs in each sample) to obtain statistically significant genetic voxels. Voxels that were significant in both samples were considered to have a 'cross-validated significance'. This procedure was repeated 100 times, and the proportion of cross-validated significance (the probability of being significant in both two independent datasets) was calculated for each category-selective voxel. The majority of voxels that were initially reported as genetic voxels (voxels marked red in the top row; see also **Figure 2b**) showed a high proportion of cross-validated significance (voxels marked cyan in the bottom row), confirming that the genetic effect in those voxels was reliable. All cross-validated genetic voxels had overlap with initially reported genetic voxels. The minimum threshold for the proportion of cross-validated significance (0.02 in the color scale bar) was obtained via the following procedure. In a control test, the pairing of twins was randomly shuffled in MZ and DZ groups so that a twin and the co-twin in each twin pair became unrelated. The

cross-validation analysis was performed based on 100 random splits of shuffled data. Only few voxels, which were scattered throughout the category-selective areas, showed cross-validated significance at a low proportion of 0.01-0.02.

Supplementary Figure 7: Pairwise Post-Hoc tests comparing the genetic effect (A) across regions. Left panel: comparisons between category-selective networks. Middle panel: comparisons between face areas. Right panel: comparisons between place areas. The Post-Hoc analysis was based on Tukey's HSD test, which was applied after running one-way ANOVA. ANOVA was not significant in body areas. Yellow indicates a significant ($p < 0.05$) comparison. Blue indicates a non-significant ($p > 0.05$) comparison.

Supplementary Figure 8: Similarity of registration-induced distortion/deformation maps in MZ and DZ twins. During the HCP analysis pipeline, the surfaces of each subject in the native space were transformed into a standard mesh template (fs_lr) through a spherical registration. For this transformation, three types of distortion maps were obtained: (i) 3D displacement maps, displacement/shift in the 3D coordinates of each vertex, (ii) isotropic distortion maps, local change in the size of vertex areas in the triangular mesh, (iii) anisotropic distortion (strain) maps, local change in the aspect ratio of vertex areas in the triangular mesh. In both isotropic and anisotropic maps, distortion for a vertex area was calculated as log(Area_Distorted/Area_Original). For each vertex, an average distortion was obtained by averaging distortions across triangles sharing the vertex. (a) In each twin pair, the 3D displacement and areal distortion maps of one twin and the co-twin were correlated using Pearson correlation. All vertices from two hemispheres were included in this correlation analysis. Distribution of correlation coefficient values was plotted for all MZ and DZ twin pairs. (b) The correlation analysis was performed for genetic and non-genetic voxels within face, body, and place areas. Mean correlation was plotted for MZ and DZ twins. Mean correlation was always higher in MZ twins than DZ twins – though the difference was not significant in some comparisons. *: $p < 0.05$, **: $p < 0.005$, ***: $p < 0.0005$; pooled t-test, Bonferroni-corrected for 3 comparisons in panel b.

Supplementary Figure 9: Category-selective voxels based on MSMSulc vs. MSMAll registrations.

Supplementary Reference:

Tsagkrasoulis D, Hysi P, Spector T, Montana G. Heritability maps of human face morphology through large-scale automated three-dimensional phenotyping. Sci Rep. 2017 Apr 19;7:45885.

Supplementary Figure 1

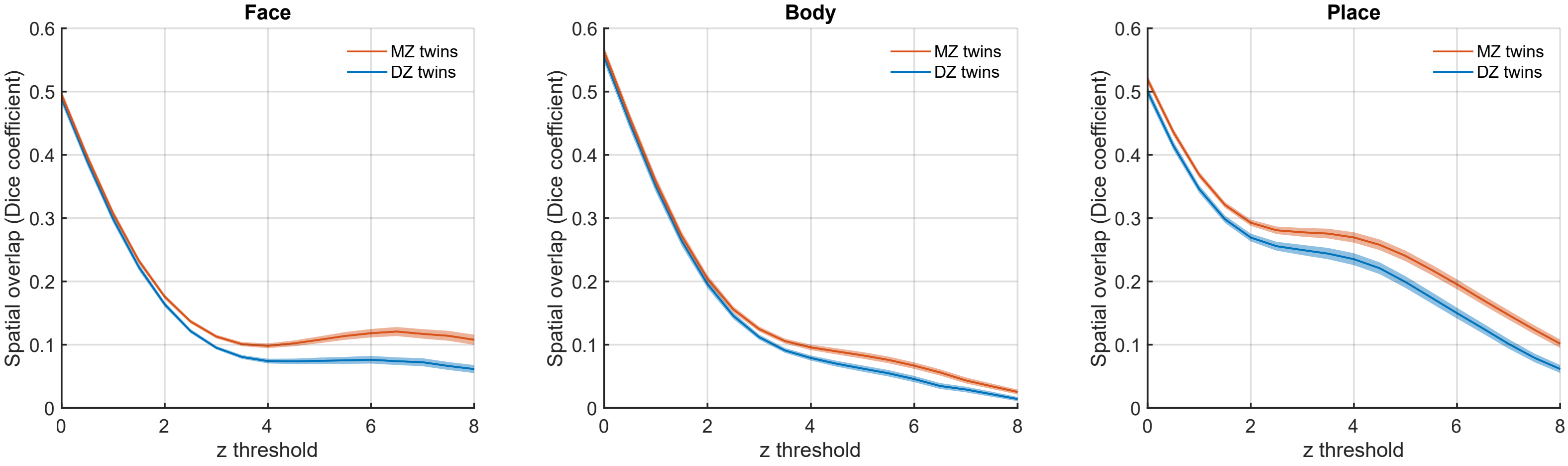

Supplementary Figure 2

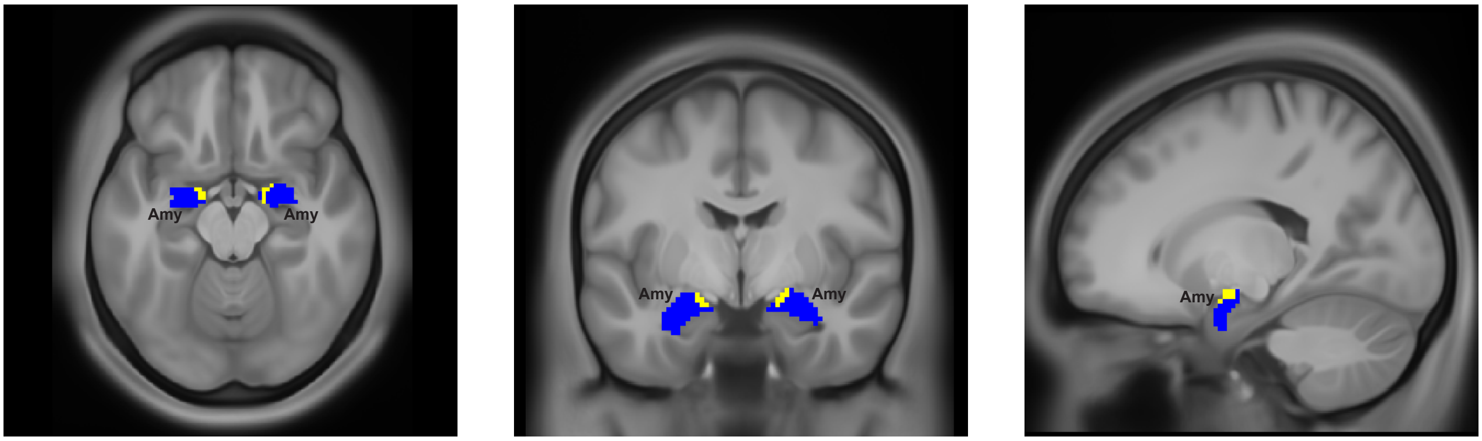

Supplementary Figure 3

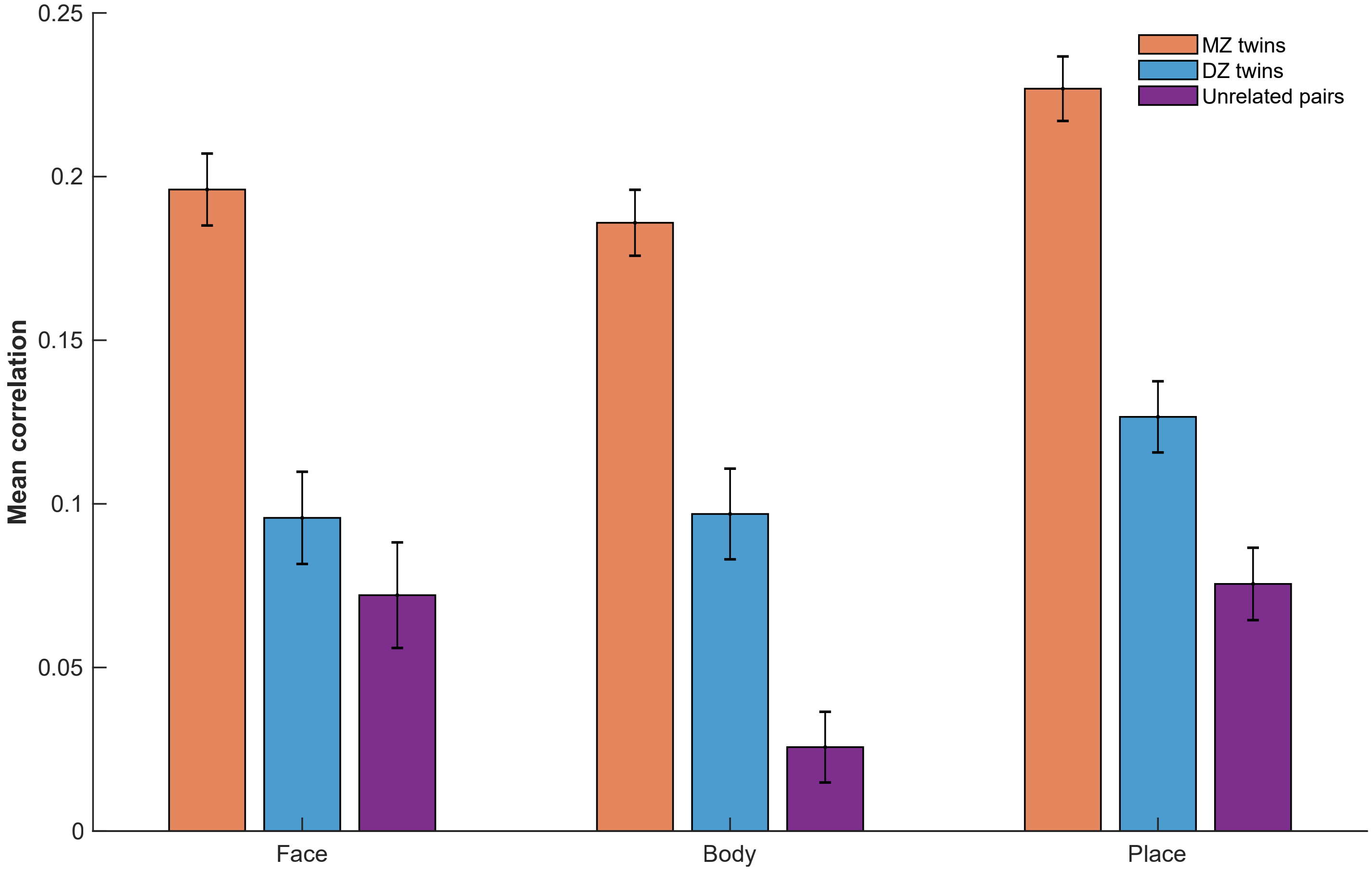

Supplementary Figure 4

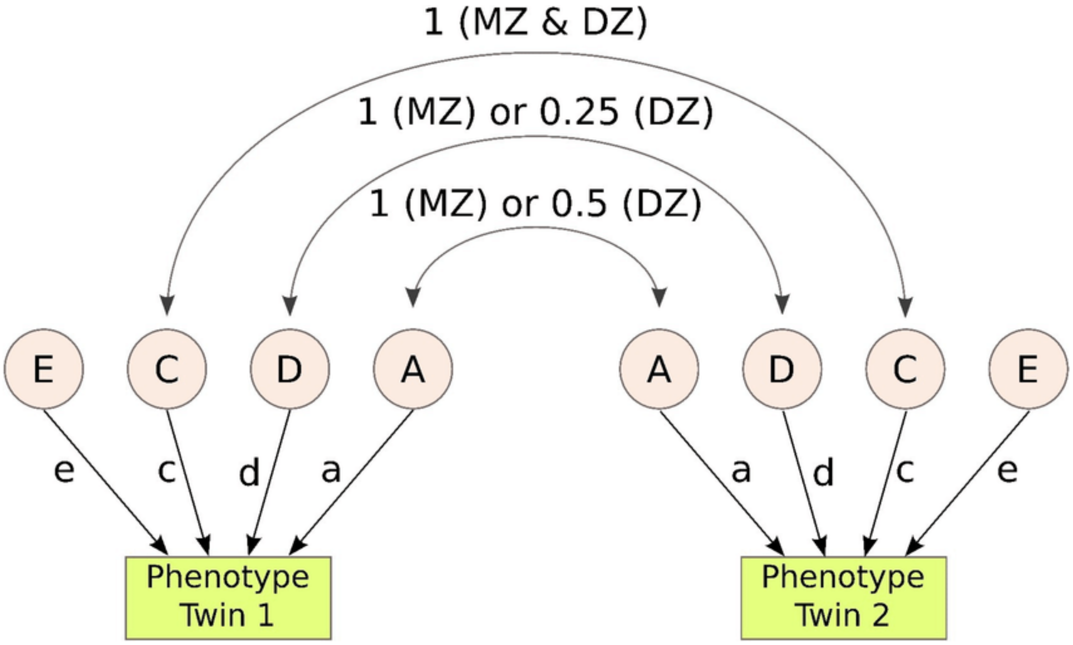

Supplementary Figure 5

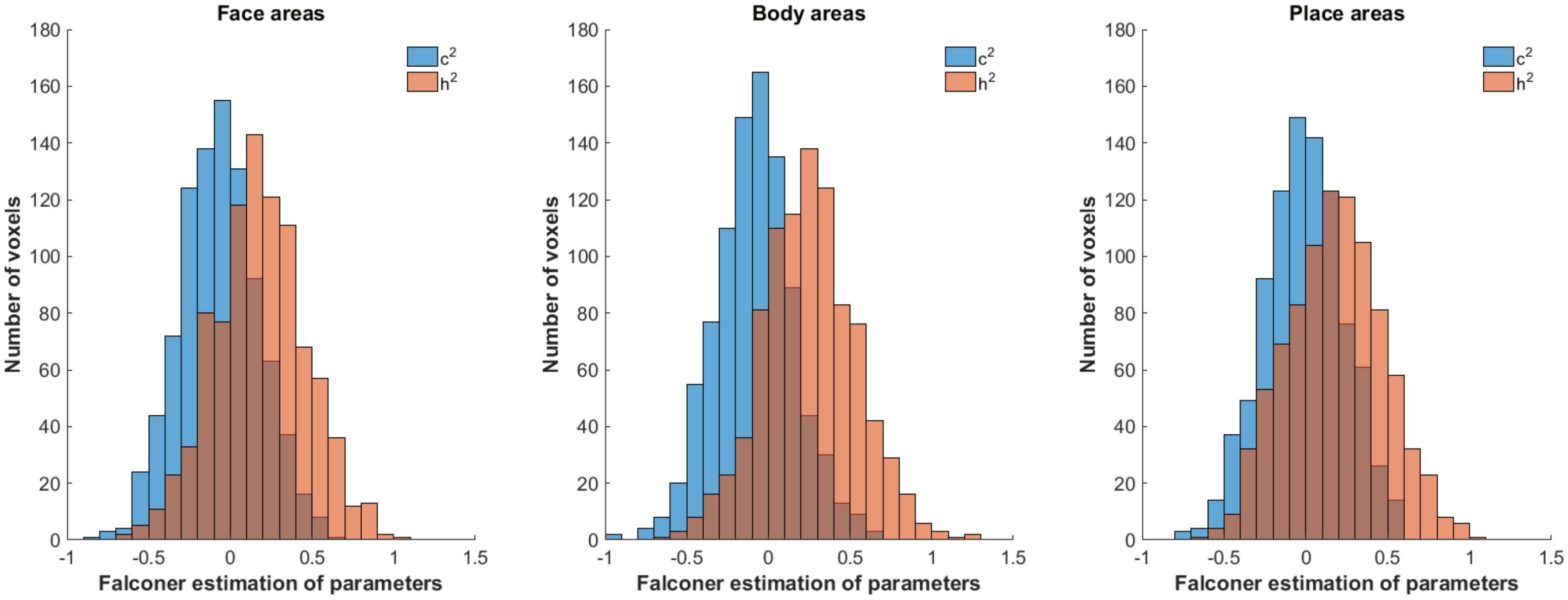

Supplementary Figure 6

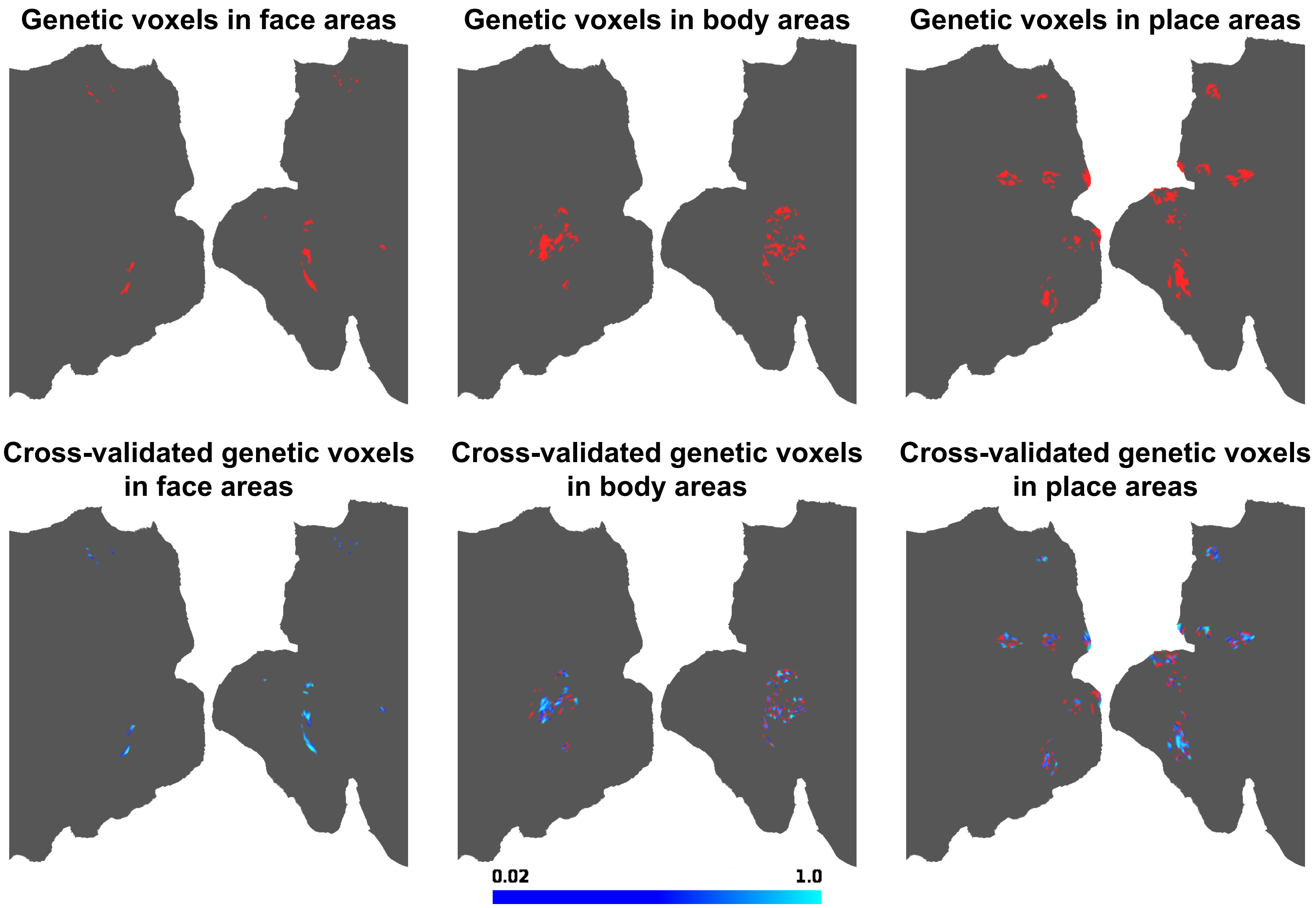

Supplementary Figure 7

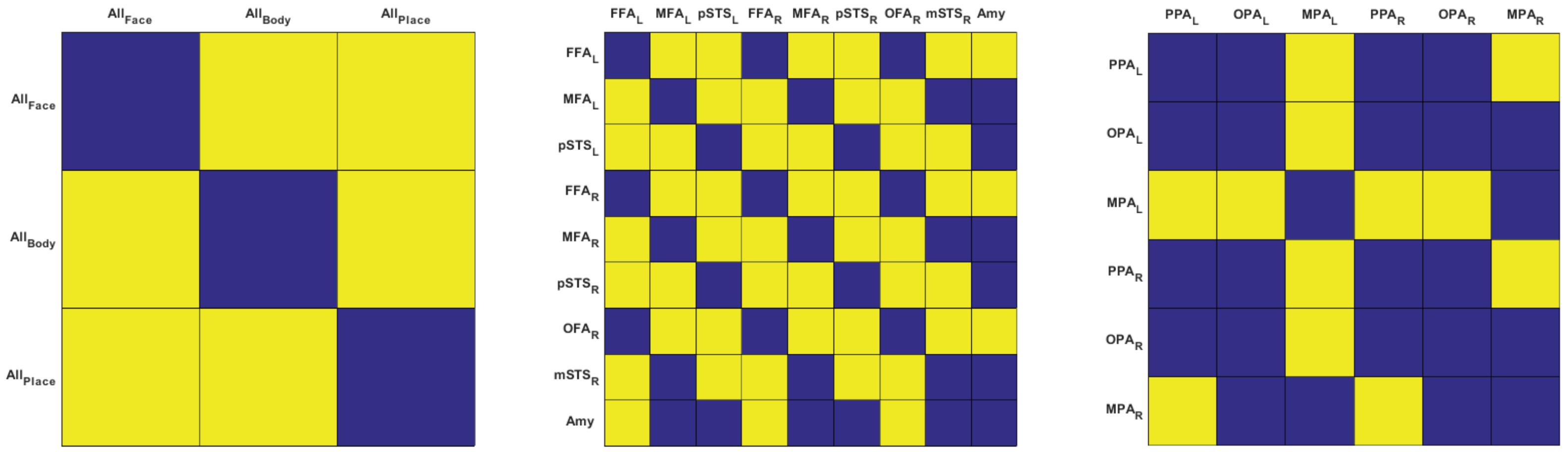

Supplementary Figure 8

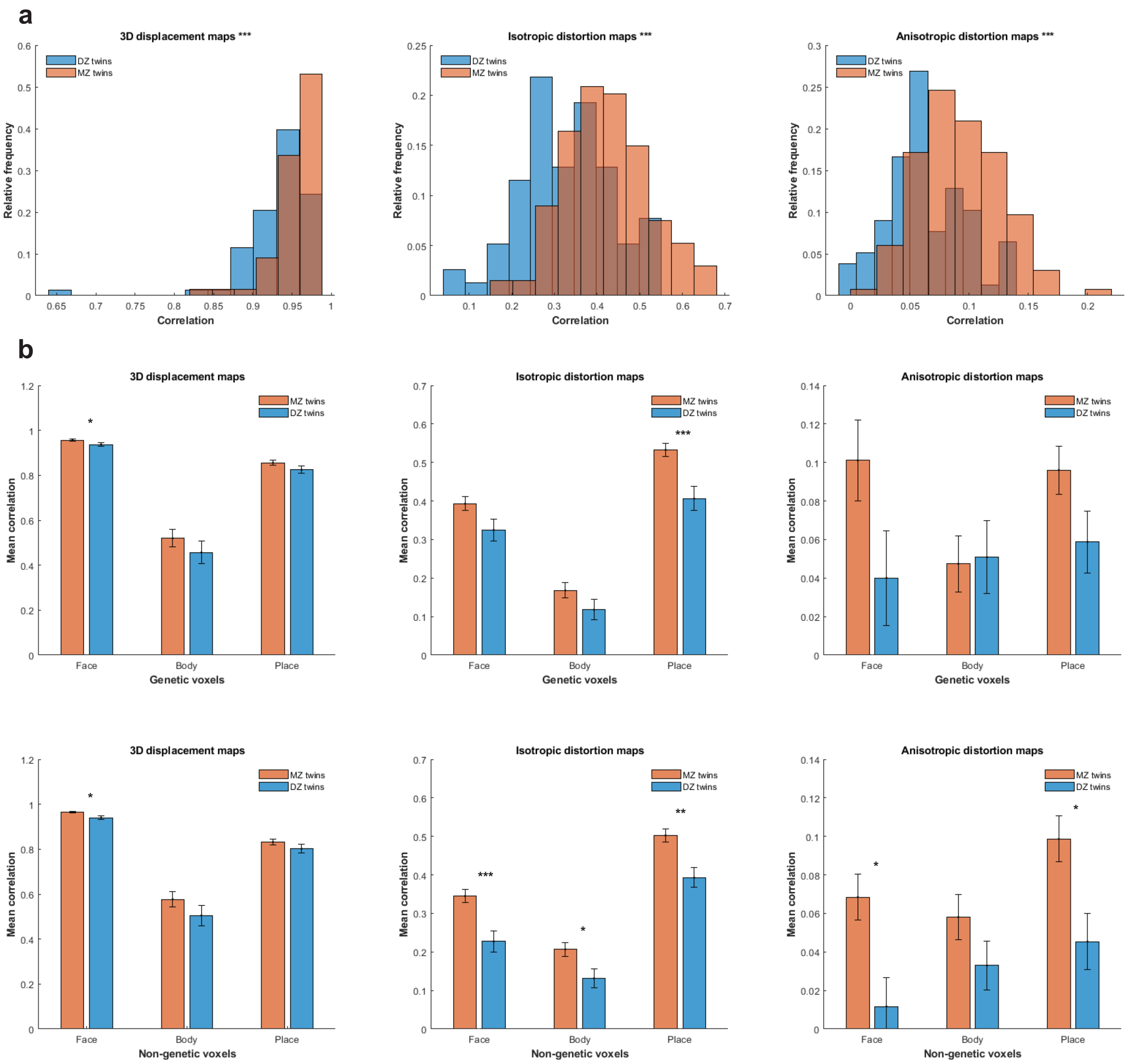

Supplementary Figure 9

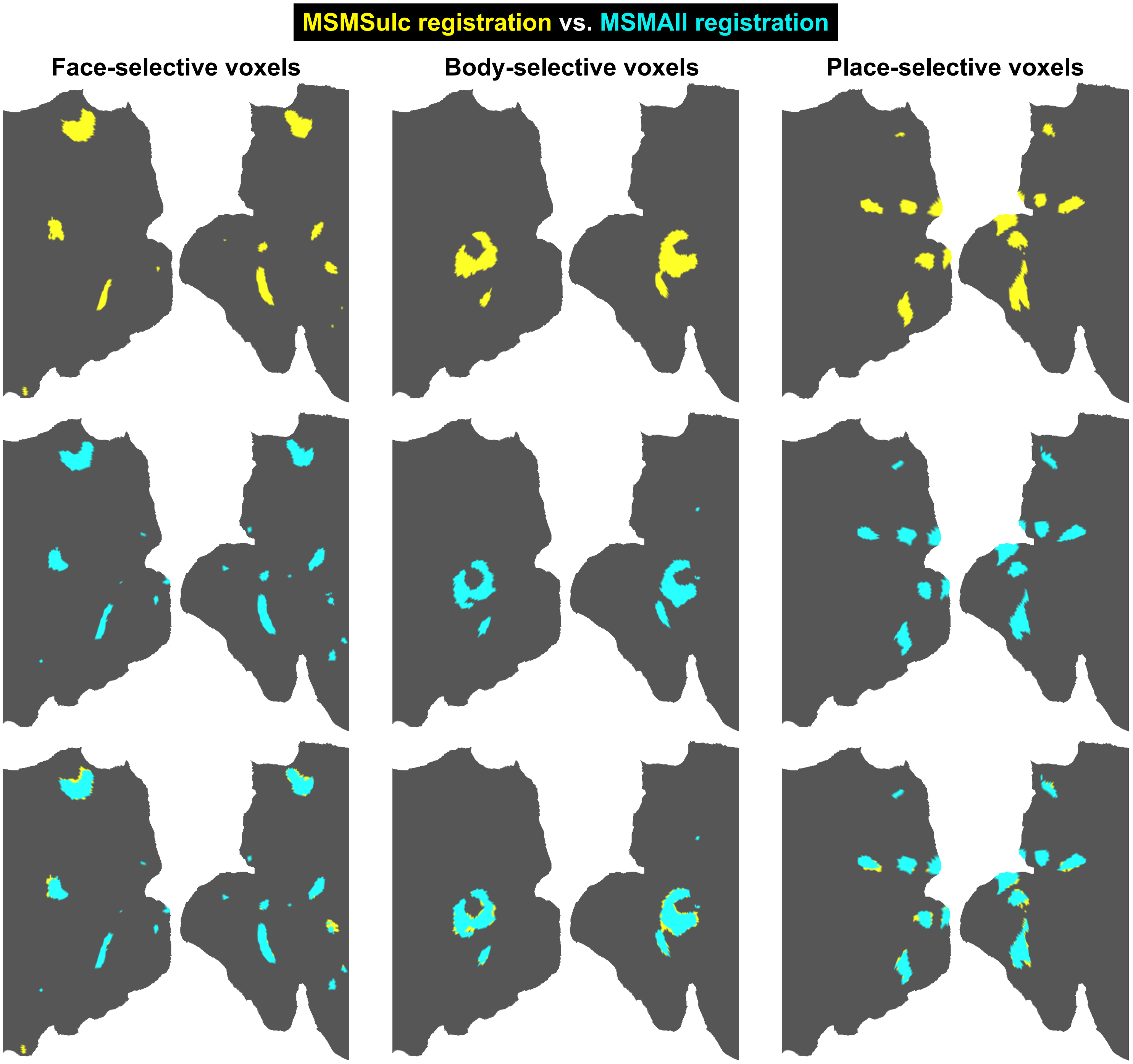

Supplement: Supplementary file 1 — Supplementary Information [file 41467_2020_14610_MOESM1_ESM.pdf]
