# Supplementary material for: Genetic influence is linked to cortical morphology in category-selective areas of visual cortex
Source: Nat Commun. 2020 Feb 5;11:709. doi: 10.1038/s41467-020-14610-8 (PMC7002610; doi:10.1038/s41467-020-14610-8)
Supplement: Supplementary file 3 — Reporting Summary [file 41467_2020_14610_MOESM3_ESM.pdf]

nature research

Corresponding author(s): Reza Rajimehr

Last updated by author(s): May 28, 2019

Reporting Summary

Nature Research wishes to improve the reproducibility of the work that we publish. This form provides structure for consistency and transparency in reporting. For further information on Nature Research policies, see Authors & Referees and the Editorial Policy Checklist .

Statistics

For all statistical analyses, confirm that the following items are present in the figure legend, table legend, main text, or Methods section.

n/a	Confirmed	
☐	☒	The exact sample size (*n*) for each experimental group/condition, given as a discrete number and unit of measurement
☐	☒	A statement on whether measurements were taken from distinct samples or whether the same sample was measured repeatedly
☐	☒	The statistical test(s) used AND whether they are one- or two-sided<br>*Only common tests should be described solely by name; describe more complex techniques in the Methods section.*
☐	☒	A description of all covariates tested
☐	☒	A description of any assumptions or corrections, such as tests of normality and adjustment for multiple comparisons
☐	☒	A full description of the statistical parameters including central tendency (e.g. means) or other basic estimates (e.g. regression coefficient) AND variation (e.g. standard deviation) or associated estimates of uncertainty (e.g. confidence intervals)
☐	☒	For null hypothesis testing, the test statistic (e.g. *F*, *t*, *r*) with confidence intervals, effect sizes, degrees of freedom and *P* value noted<br>*Give P values as exact values whenever suitable.*
☒	☐	For Bayesian analysis, information on the choice of priors and Markov chain Monte Carlo settings
☒	☐	For hierarchical and complex designs, identification of the appropriate level for tests and full reporting of outcomes
☐	☒	Estimates of effect sizes (e.g. Cohen's *d*, Pearson's *r*), indicating how they were calculated

Our web collection on statistics for biologists contains articles on many of the points above.

Software and code

Policy information about availability of computer code

Data collection	E-Prime
Data analysis	Workbench, Matlab, R, OpenMx

For manuscripts utilizing custom algorithms or software that are central to the research but not yet described in published literature, software must be made available to editors/reviewers. We strongly encourage code deposition in a community repository (e.g. GitHub). See the Nature Research guidelines for submitting code & software for further information.

Data

Policy information about availability of data

All manuscripts must include a data availability statement. This statement should provide the following information, where applicable:

- Accession codes, unique identifiers, or web links for publicly available datasets
- A list of figures that have associated raw data
- A description of any restrictions on data availability

All data used in this manuscript are part of publicly available and anonymized HCP database (https://www.humanconnectome.org). All analysis codes are available for sharing upon request.

Field-specific reporting

Please select the one below that is the best fit for your research. If you are not sure, read the appropriate sections before making your selection.

☒ Life sciences ☐ Behavioural & social sciences ☐ Ecological, evolutionary & environmental sciences

Life sciences study design

All studies must disclose on these points even when the disclosure is negative.

Sample size	All twin subjects from the HCP database (n = 424) were used in this study.
Data exclusions	No data were excluded from the analyses.
Replication	A cross-validation analysis was performed (see Supplementary Figure 4).
Randomization	Blocks of four visual categories were presented randomly to the subjects during the fMRI task.
Blinding	The MZ and DZ twin pairs were defined based on a genetic test.

Reporting for specific materials, systems and methods

We require information from authors about some types of materials, experimental systems and methods used in many studies. Here, indicate whether each material, system or method listed is relevant to your study. If you are not sure if a list item applies to your research, read the appropriate section before selecting a response.

Materials & experimental systems

n/a	Involved in the study	
☒	☐	Antibodies
☒	☐	Eukaryotic cell lines
☒	☐	Palaeontology
☒	☐	Animals and other organisms
☐	☒	Human research participants
☒	☐	Clinical data

Methods

n/a	Involved in the study	
☒	☐	ChIP-seq
☒	☐	Flow cytometry
☐	☒	MRI-based neuroimaging

Human research participants

Policy information about studies involving human research participants

Population characteristics	In this study, we used the “HCP1200” dataset (March 2017 data release) of healthy adults aged 22-35 (https://www.humanconnectome.org/study/hcp-young-adult/document/1200-subjects-data-release). The dataset included 424 twin subjects (252 females, 172 males). Of 212 twin pairs, 134 pairs were genetically-confirmed MZ twins and 78 pairs were genetically-confirmed, same-sex DZ twins.
Recruitment	Subjects were recruited from Washington University (St. Louis, MO) and the surrounding area.
Ethics oversight	The HCP data were acquired using protocols approved by the Washington University institutional review board. Written informed consent was obtained from all subjects.

Note that full information on the approval of the study protocol must also be provided in the manuscript.

Magnetic resonance imaging

Experimental design

Design type	Task fMRI, block design
Design specifications	Subjects performed 2 runs of the working memory task. Each run contained 8 task blocks (25 s each) and 4 fixation blocks (15 s each). The 4 different stimulus types (faces, places, tools, and body parts) were presented in separate task blocks. Each task block contained 10 trials. On each trial, the stimulus was presented for 2 s, followed by a 500 ms inter-trial interval. Within each run, 4 blocks used a 2-back working memory task (respond ‘target’ whenever the current stimulus was the same as the one two back) and the other 4 blocks used a 0-back working memory task (respond ‘target’ whenever the current stimulus was the same as the target stimulus presented at the start of the block). A 2.5 s cue indicated the task type (and target for 0-back) at the start of the block. In each block, there were 2 targets and 2–3 non-target stimuli (repeated items in the wrong n-back position, either 1-back or 3-back).
Behavioral performance measures	Proportion of correct button presses

Acquisition

Imaging type(s)	functional and structural MRI
Field strength	3T Siemens scanner
Sequence & imaging parameters	3D T1w MPRAGE and 3D T2w SPACE sequences at 0.7 mm isotropic resolution<br>Multi-band EPI sequence with parameters of TR=720 ms, 2 mm isotropic voxels, and multi-band acceleration factor of 8<br>Spin echo field map sequence
Area of acquisition	Whole-brain

Diffusion MRI ☐ Used ☒ Not used

Preprocessing

Preprocessing software	Data were preprocessed and analyzed using the publicly released HCP pipelines. The software packages used for analysis included Connectome Workbench commandline tools, FreeSurfer, and FSL. Connectome Workbench 'wb_view' GUI (http://www.humanconnectome.org/software/connectome-workbench.html) was used for visualization of maps and creating ROIs.
Normalization	Data from the cortical gray matter ribbon were projected onto the surface and then onto the standard grayordinates space. Subcortical data were also projected to a set of subcortical gray matter structures in the grayordinates space. Data were minimally smoothed by a 2mm FWHM Gaussian kernel in the grayordinates space.
Normalization template	The standard 'CIFTI grayordinates' space (91,282 vertices/voxels with ~ 2 mm cortical vertex spacing and 2 mm isotropic subcortical voxels)
Noise and artifact removal	Data were cleaned up for artifacts and structured noise using ICA+FIX.
Volume censoring	No volume censoring was performed.

Statistical modeling & inference

Model type and settings	Univariate analysis. For the working memory task, 8 regressors/predictors were used in the GLM design – one for each type of stimulus in each of the N-back conditions.
Effect(s) tested	Linear contrasts were computed to estimate effects of interest: each stimulus type versus all others, collapsing across memory load. Fixed-effects analyses were conducted to estimate the average effects across runs within each subject, then mixed-effects analyses treating subjects as random effects were conducted to obtain group-average maps.

Specify type of analysis: ☐ Whole brain ☐ ROI-based ☒ Both

Anatomical location(s)	The category-selective voxels were defined as the top 1% of voxels (913 out of 91,282 voxels) which had the highest z values in a given contrast (e.g. faces vs. all other categories). The 99th percentile corresponded to the cutoff-point z values of 12.38, 16.89, and 27.35 in group-average face, body, and place maps, respectively.
Statistic type for inference (See Eklund et al. 2016)	Voxel-wise
Correction	FDR correction

Models & analysis

n/a	Involved in the study
☒	☐ Functional and/or effective connectivity
☒	☐ Graph analysis
☒	☐ Multivariate modeling or predictive analysis
